# Beyond volumetry: Considering age-related changes in brain shape complexity using fractal dimensionality

**DOI:** 10.1016/j.nbas.2021.100016

**Published:** 2021-05-28

**Authors:** Christopher R. Madan

**Affiliations:** School of Psychology, University of Nottingham, Nottingham, UK

**Keywords:** Structural complexity, Atrophy, Gyrification, Cortical folding, Brain morphometry, Ventricles

## Abstract

Gray matter volume for cortical, subcortical, and ventricles all vary with age. However, these volumetric changes do not happen on their own, there are also age-related changes in cortical folding and other measures of brain shape. Fractal dimensionality has emerged as a more sensitive measure of brain structure, capturing both volumetric and shape-related differences. For subcortical structures it is readily apparent that segmented structures do not differ in volume in isolation—adjacent regions must also vary in shape. Fractal dimensionality here also appears to be more sensitive to these age-related differences than volume. Given these differences in structure are quite prominent in structure, caution should be used when examining comparisons across age in brain function measures, as standard normalisation methods are not robust enough to adjust for these inter-individual differences in cortical structure.

## 1 Introduction

Brain structure and function change in many ways due to aging. Conventional measures of brain structure are primarily volumetric—regional cortical and subcortical volume, though cortical volume can be further decomposed into thickness and surface area [25], [20]. While these measures are useful, they have limitations in sensitivity to structural changes that are complemented by the approaches that measure shape-related properties.

**Fig. 1:**
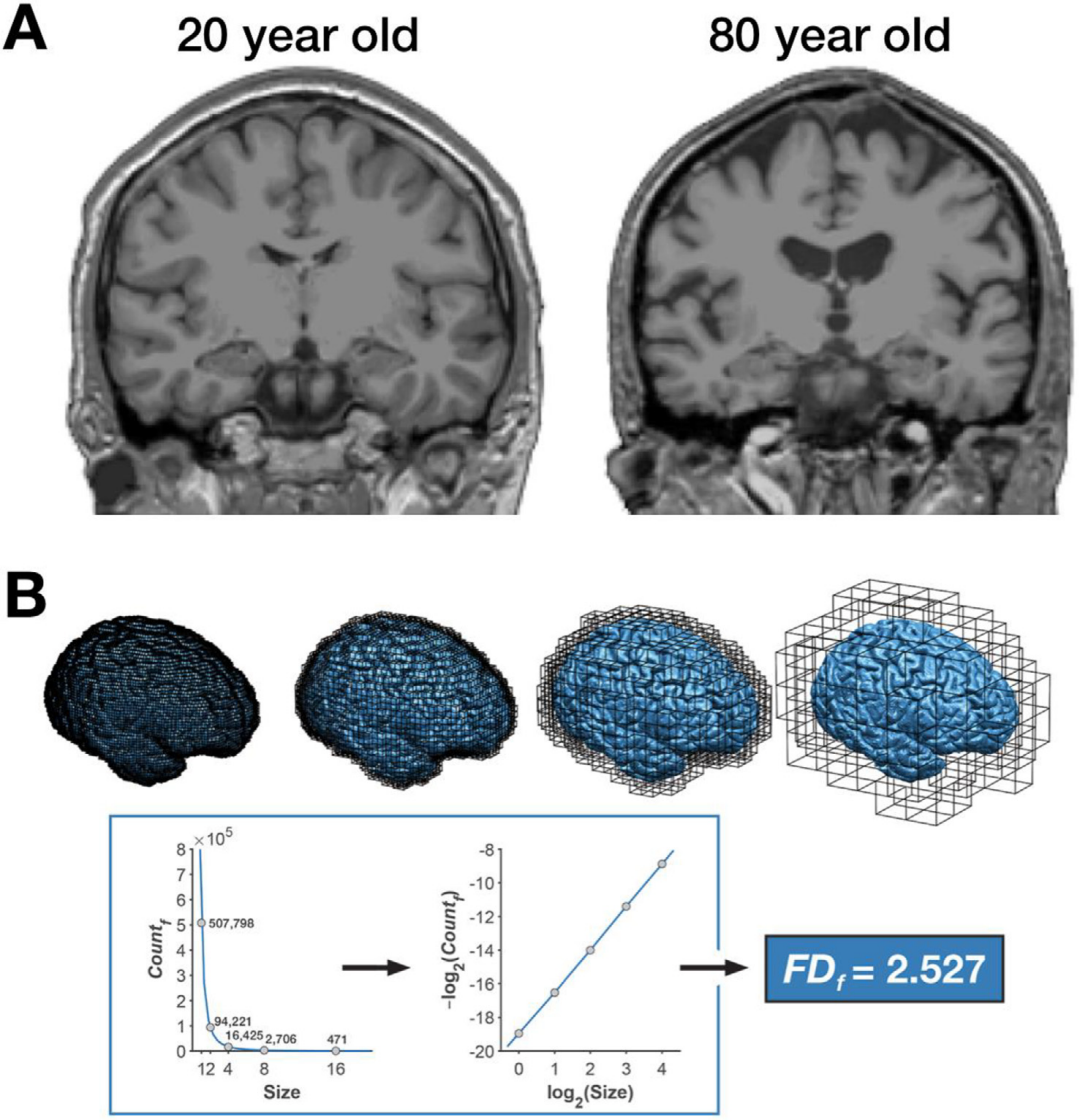
(A) T1-weighted coronal sections of individuals that are 20 and 80 year old (left and right, respectively). (B) Illustration of the fractal dimensionality calculation. This calculation involves counting the volume—in isotropic voxels—of a structure, here shown as coronal grey matter, across multiple spatial scales. Here this was done at 1, 2, 4, 8, and 16 mm isotopic voxels, including any voxel that includes the structure (i.e., any voxel with partial volume is counted). These counts and voxel sizes are then log–log transformed and the slope of the resulting line is the fractal dimensionality value of the structure.

When visually examining T1-weighted volumes that vary in age, as in Fig. 1A, cortical thinning is not apparent. Rather, there are clear differences in cortical folding, ventricular enlargement, and subcortical shrinkage. Indeed, these are the qualitative features assessed in ratings of atrophy [3], [22]. Quantification of gyrification based on comparing surface area versus a smoothed outer contour has been used in early work [29] and has been refined to take advantage of modern three-dimensional reconstruction methods [26]. Gyrification has been shown to be sensitive to age-related differences in cross-sectional samples [6], [15], [18], [1]. More recently, however, sulcal morphology—width and depth—appear to be more direct measures of longitudinal changes in cortical folding [13]. Sulcal morphology has also previously been shown to be sensitive to aging [8], [9], [11].

Fractal dimensionality is a scale-invariant mathematical measure of shape complexity. While not specific to brain structure, it has been used in the neuroimaging literature for decades [4], [27], [7]. Most studies have focused on differences in patients and the use of fractal dimensionality to understand aging has only recently emerged [15], [16], [19]. Here it has been shown that for cortex, fractal dimensionality is more sensitive to age-related differences than cortical thickness or gyrification [15], [18]. Briefly, fractal dimensionality involves counting the number of voxels that include a structure at an initial spatial scale (e.g., 1-mm isotropic) and then using coarser spatial scales and again counting the voxels that include the structure, as shown in Fig. 1B. For simple structures such as a cube or sphere, the shape is still apparent even if voxel resolution becomes coarse. In contrast, for a complex structure such as a human brain, lower resolution volumes lose details of the shape; fractal dimensionality is a measure of this loss in fidelity (see [15]. Fractal dimensionality does not require high-field MRI and 1-mm isotropic voxels are often sufficient, but higher-resolution data is necessary for smaller structures (e.g., hippocampal subfields).

If shape was independent of volume and unrelated to age, fractal dimensionality should provide no advantages as it is a measure of shape complexity. However, if shape and volume were linked, fractal dimensionality would be more sensitive than volumetric measures. As an example of this we can consider that age-related differences in subcortical volume have been well established [24], [28]. Should we assume that the shape of a structure simply ‘scales’ in space as it varies in volume, or that there would also be associated changes in shape complexity? Morever, we should further expect related variations on adjacent structures, these are segmented structures with other brain tissues surrounding them. Indeed, age-related changes in fractal dimensionality of subcortical structures are more pronounced than those in volume—though this is not true of the ventricles [16], [12]. As an additional benefit, relying on shape properties allows fractal dimensionality to be more resilient to measurement error, such as head position across multiple sessions [17] or within-session head motion [10].

Recent advances provide strong evidence that aging is not only reflected in volumetric differences. This approach is ripe for further research, particularly in relating these structural differences to behaviour, other modalities, and other phenotypic measures more broadly. For instance, the functional implications of inter-individual differences in tertiary sulci [21] and cortical myelination [2] are poorly understood. Given the richness of available open-access data, we now have the ability to conduct complex analyses on tens of hours of task-related MRI data from a small sample of highly characterised participants, minimising structural variability as a source of variance [5], [23], [14]. Specifically, current normalisation methods for transforming from native space to standardised space (e.g., MNI) are not sufficiently considerate of inter-individual differences in cortical folding. As a field we should carefully consider when these approaches may be more informative than collecting a small amount of functional data from hundreds of individuals.
